# Enhanced Electrocatalytic Activity for Water Splitting on NiO/Ni/Carbon Fiber Paper

**DOI:** 10.3390/ma10010015

**Published:** 2016-12-28

**Authors:** Ruoyu Zhang, Hehe Wei, Wenjie Si, Gang Ou, Chunsong Zhao, Mingjun Song, Cheng Zhang, Hui Wu

**Affiliations:** 1 School of Materials Science and Engineering, Shanghai Institute of Technology, Shanghai 201418, China; 156081128@mail.sit.edu.cn; 2 State Key Laboratory of New Ceramics and Fine Processing, School of Materials Science and Engineering, Tsinghua University, Beijing 100084, China; weihh15@mails.tsinghua.edu.cn (H.W.); wjsi@tsinghua.edu.cn (W.S.); ougang@mail.tsinghua.edu.cn (G.O.); zhaocs14@mails.tsinghua.edu.cn (C.Z.); songmj13@mails.tsinghua.edu.cn (M.S.); huiwu@mail.tsinghua.edu.cn (H.W.); 3 Department of Chemistry and Collaborative Innovation Center for Nanomaterial Science and Engineering, Tsinghua University, Beijing 100084, China

**Keywords:** NiO/Ni nanoparticles, hydrogen evolution reaction, electrocatalyst

## Abstract

Large-scale growth of low-cost, efficient, and durable non-noble metal-based electrocatalysts for water splitting is crucial for future renewable energy systems. Atomic layer deposition (ALD) provides a promising route for depositing uniform thin coatings of electrocatalysts, which are useful in many technologies, including the splitting of water. In this communication, we report the growth of a NiO/Ni catalyst directly on carbon fiber paper by atomic layer deposition and report subsequent reduction and oxidation annealing treatments. The 10–20 nm NiO/Ni nanoparticle catalysts can reach a current density of 10 mA·cm^−2^ at an overpotential of 189 mV for hydrogen evolution reactions and 257 mV for oxygen evolution reactions with high stability. We further successfully achieved a water splitting current density of 10 mA·cm^−2^ at 1.78 V using a typical NiO/Ni coated carbon fiber paper two-electrode setup. The results suggest that nanoparticulate NiO/Ni is an active, stable, and noble-metal-free electrocatalyst, which facilitates a method for future water splitting applications.

## 1. Introduction

Electrochemical water splitting has been an efficient approach to produce hydrogen, which is a clean alternative to fossil fuels. Hydrogen evolution reaction (HER), a half reaction of water splitting, is significantly crucial to hydrogen evolution. To this end, tremendous efforts have been devoted to highly active catalysts for hydrogen evolution reaction using transition metals and their compounds [1,2,3,4,5]. Among them, Ni-based nanomaterials have attracted growing interest as hydrogen evolution reaction catalysts [6,7,8,9], owing to their many excellent properties, which include chemical stability, earth abundance, and excellent electrochemical property [10]. For example, partially reduced nickel interfaced with nickel oxide from the decomposition of Ni(OH)_2_ exhibited highly active hydrogen evolution reaction activity [8]. Hierarchically multifunctional porous NiSx provided nickel sulfide-active sites and results in effective water splitting electrocatalysts for hydrogen evolution reaction [11]. Yao obtained isolated Ni-atom/graphitic carbon with an active exchange current density of 1.2 mA·cm^−2^ [12]. Partial oxidation of a 2D ultrathin nickel nanosheet array lead to the exhibition of superior HER performance [13]. However the production of efficient and durable Ni-based electrocatalyst for HER still remains a challenge.

Herein we report on the direct loading of NiO/Ni nanoparticles on carbon fiber paper, based on atomic layer deposition and followed by reduction-oxidation annealing treatment. Compared with other electrocatalysts, the NiO/Ni nanoparticles loaded on carbon fiber paper not only exhibit significantly enhanced HER activity and impressive durability, but can also be regarded as cathode and anode in a two-electrode system for water splitting. For example, the NiO/Ni nanoparticles loaded on carbon fiber paper exhibit overpotential for the current density of 10 mA·cm^−2^ at 189 mV, for hydrogen evolution reaction, and 257 mV for oxygen evolution reaction. The results indicate that the NiO/Ni catalyst loaded on carbon fiber paper exhibits active and stable electrocatalytic activity towards a hydrogen evolution reaction and an oxygen evolution reaction. Furthermore the facile synthetic procedure of a NiO/Ni catalyst would open up a promising way to achieve more efficient and more active water splitting for hydrogen evolution reactions.

## 2. Experiment

NiO was deposited on the TORAY carbon paper (0.19 mm, 0.44 g·cm^−3^, TGP-H-060, Toray, Tokyo, Japan), supported by atomic layer deposition (QinALD, Institute of Coal Chemistry Chinese Academy of Sciences, Taiyuan, China). During the process of deposition; Ni(Cp)_2_ and O_3_ were used as Ni and O precursors, respectively. Ni(Cp)_2_ was evaporated at 80 °C. The flow rate of O_3_ was 15 sccm. High purity nitrogen gas was used as the carrier gas with a flow rate of 50 sccm. The growth temperature in the ALD process was 270 °C. Typically an ALD growth cycle for NiO was 6 s Ni(Cp)_2_ pulse, 12 s N_2_ purge, 25 s waiting time, 1 s O_3_ pulse, 12 s N_2_ purge, and 25 s waiting time. 300 cycles were deposited on carbon paper; each was about 10 nm. The as-deposited samples were regarded as the ALD-NiO/C sample. The as-deposited NiO/C samples were annealed at different annealing temperatures; from 300 to 600 °C under a 90% Ar and 10% H_2_ atmosphere for 2 h. These samples were denoted as Ni/C samples. The as-annealed NiO/C samples under the Ar and H_2_ atomosphere were than annealed at 200 °C under an air atmosphere for 30 min, in order to slightly oxidize the already reduced NiO/C samples.

X-ray diffraction patterns and the crystal structures were collected using X-ray diffractometer (XRD, D/max 2500V, Rigaku, Tokyo, Japan) with a Cu target. The SEM images were obtained using a field-emission scanning electron microscope (FESEM, JSM-6701F, JEOL, Beijing, China) with an operated accelerating voltage of 15 kV. Transmission electron microscopic (TEM, JEOL) images were measured by a JEOL-2010 and a TecnaiTF20 (FEI, Hillsborough, CA, USA) microscope. X-ray photoelectron spectroscopy measurements for NiO/C samples were performed using an X-ray photoelectron spectrometer (XPS, Escalab 250Xi, Thermo Fisher Scientific, Boston, MA, USA) equipped with an Al Kα radiation source (1487.6 eV) and hemispherical analyzer with a pass energy of 30.0 eV and an energy step size of 0.05 eV. The collected data were corrected for charging effect-induced peak shifts using the binding energy (BE) of C 1*s* peak of substrate (284.8 eV). Spectral deconvolution was performed by Shirley background subtraction using a Voigt function, convoluting the Gaussian and Lorentzian functions.

Electrochemical measurements were performed with a conventional three electrode system; NiO/C as a working electrode, Ni foam as a counter electrode, and Ag/AgCl as a reference electrode by using an Electrochemical Workstation (CHI660E, Shanghai Chenhua Instrument Corporation, Shanghai, China). In a hydrogen evolution reaction measurement, a linear sweep voltammetry from −1 to −1.8 V with scan rate of 5 mV·s^−1^ was conducted in a 1 M KOH solution. Potential vs. Ag/AgCl was converted to Potential vs. Reversible hydrogen electrode (RHE) using the relationship of *E*(RHE) = *E*(Ag/AgCl) + 0.198 + 0.059 × pH. A stability test was performed by holding the working electrode at 270 mV of overpotential vs. RHE for 10 h. 

## 3. Results and Discussion

**Figure 1:**
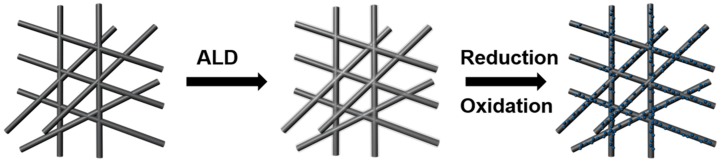
The schematic outline for the NiO/Ni nanoparticles deposited on carbon fiber paper. ALD: atomic layer deposition.

**Figure 2:**
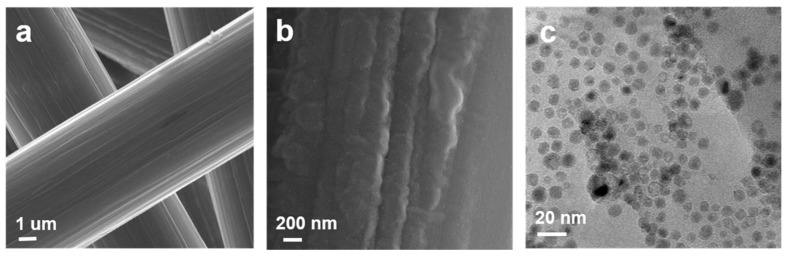
Microstructural of NiO/Ni nanoparticles loaded on carbon fiber paper. (**a**,**b**) Scanning electron microscopic (SEM) images; and (**c**) transmission electron microscopic (TEM) image.

The schematic diagram of NiO/Ni nanoparticles loaded on carbon fiber paper (NiO/Ni/C) is shown in Figure 1. The NiO film was deposited on carbon fiber paper (NiO/C) using the ALD technique. Followed by a reduction annealing process at 500 °C in an H_2_/Ar mixed atmosphere for 2 h, the NiO/C samples were annealling-treated at 200 °C for 30 min. This synthesis procedure is compatible with sizable electrodes; for example, Figure S1 presents a 1 cm × 1.2 cm digital photograph of the carbon fiber paper loaded with NiO. The NiO/Ni/C sample was characterized by a scanning electron microscope and transmission electron microscope. Figure 2 illustrates the catalyst prepared on commercial carbon fiber paper. The carbon fiber papers are conformably covered with NiO/Ni nanoparticles, which are 10–20 nm in size. This is attributed to the agglomeration of NiO film during the annealing process. As shown in Figure S2, the carbon paper is well crystalized showing a clear lattice with a spacing of 0.375 nm, which corresponds to the XRD analysis (Figure S3). In the TEM image of the ALD-NiO/C sample, the measured NiO (111) d-spacing of 0.241 nm and NiO (200) d-spacing of 0.208 nm confirm the existence of the well crystalline of NiO [14], suggesting that was NiO was successfully loaded onto the carbon fiber paper during the ALD process. Compared with the ALD-NiO/C sample, the reduced NiO loaded on carbon fiber paper (Ni/C) and NiO/Ni/C samples clearly show the structure of the nanoparticles (Figure S4). The electron dispersive X-ray spectrum (EDX) also verifies the existence of NiO and Ni, in which there are only three elements of Ni, O, and C in all samples. More importantly, the NiO/Ni/C sample exhibits more remarkable nanoparticles, suggesting larger specific surface area and higher electrocatalytic activity compared to the ALD-NiO/C and Ni/C samples.

**Figure 3:**
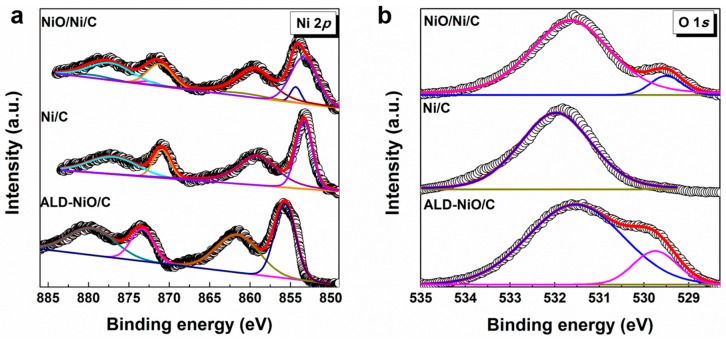
X-ray photoelectron spectrum (XPS) of ALD-NiO/C, Ni/C and NiO/Ni/C samples. (**a**) Ni 2*p*; (**b**) O 1*s*.

The chemical bonding states of the ALD-NiO/C, Ni/C and the NiO/Ni/C samples were investigated by X-ray photoelectron spectrometer (XPS). The survey XPS spectrum (Figure S5) shows Ni, O, and C elements in the ALD-NiO/C sample. As shown in Figure 3, the spectra of the ALD-NiO/C sample are fitted to four peaks, appearing at 879.6, 873.4, 861.5, and 855.7 eV and corresponding to Ni 2*p*_1/2_, the satellite, Ni 2*p*_3/2_ and the satellite, respectively [11]; this is attributed to the Ni^2+^ state for NiO. The 17.7 eV separation of the 2*p* two peaks is due to the spin-orbit interaction. After the hydrogen-annealing treatment, the Ni 2*p* XPS spectra of the Ni/C sample remain fitted to four peaks but shift towards a lower binding energy direction of about 2.5 ± 0.05 eV, corresponding to the NiO of Ni metal. It suggests that the NiO was reduced by hydrogen during the annealing process. The NiO/Ni/C sample increases by another four peaks. Specially, the eight peaks are typical of Ni–Ni and Ni–O bonds. The weak intensity of NiO peaks indicate that the Ni/C sample was oxidized partially. It suggests the NiO/Ni nanoparticles structure of the NiO/Ni/C sample. The spectra of O 1*s* for the ALD-NiO/C sample are fitted two peaks at 529.7 [15] and 531.5 eV [16], which are assigned to Ni–O and absorbed H_2_O, respectively. After the H_2_ annealing process, the Ni–O peak of O 1*s* spectra disappears, in accord with the results of Ni 2*p*. However the O 1*s* XPS spectra of the NiO/Ni/C sample have another peak compared to the Ni/C sample, which can be associated with the Ni–O bond. Moreover the intensity of Ni–O for the NiO/Ni/C sample at 530.7 eV [17] becomes weaker, indicating the slow oxidation of the Ni metal in the air conditions. All XPS results are in accord with TEM analysis in Figure 2.

**Figure 4:**
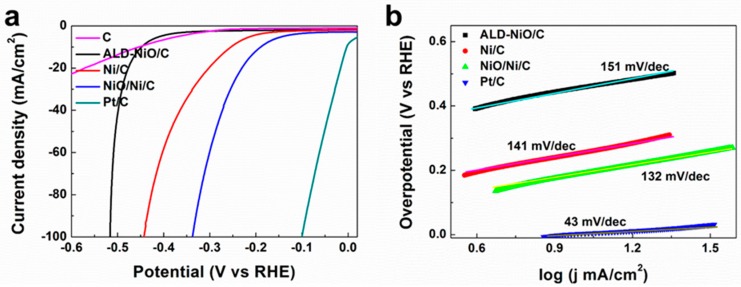

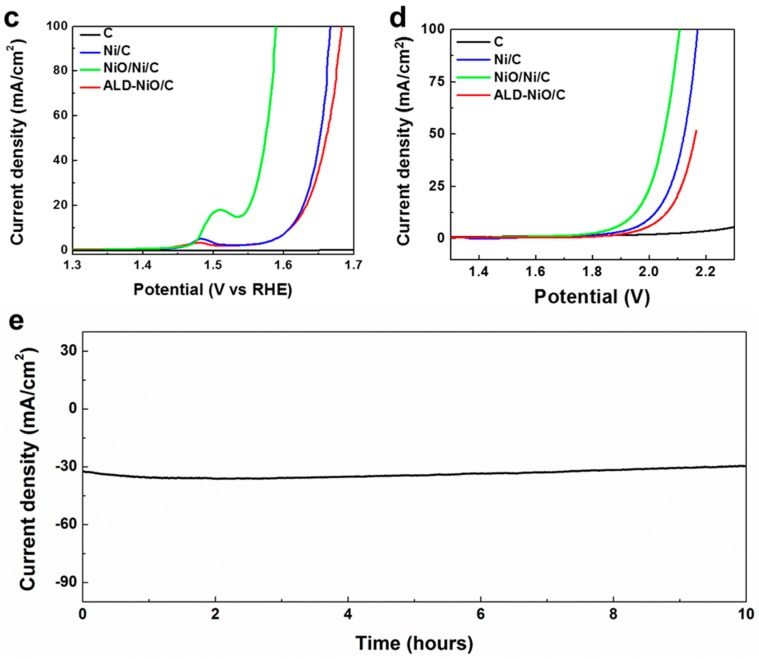
Electrocatalytic activity of ALD-NiO/C, Ni/C and NiO/Ni/C samples for water splitting in 1 M KOH; (**a**) cathodic polarization curves; (**b**) the corresponding hydrogen evolution reaction (HER) Tafel plot; (**c**) oxygen evolution reaction (OER) activity; (**d**) polarization curves for NiO/Ni/C||NiO/Ni/C, Ni/C||Ni/C, ALD-NiO/C||ALD-NiO/C, and C||C in a two-electro setup for overall water splitting; (**e**) the stability of NiO/Ni nanoparticles loaded on carbon fiber paper. All Linear Sweep Voltammetry (LSV) curves are iR corrected. iR represents internal resistance in different solutions, it is a parameter.

The electrocatalytic activity of the carbon fiber paper, the ALD-NiO/C, the Ni/C, and the NiO/Ni/C samples toward a hydrogen evolution reaction was carried out using a three-electrode electrochemical cell in 1 M KOH solution with Pt/C as the reference. The ohmic potential drop losses caused by electrolyte resistance were compensated before comparison. As shown in Figure 4, the polarization plot reveals that the NiO/Ni/C sample displays a small onset potential of 115 mV for HER activity. By contrast, the ALD-NiO/C and Ni/C samples show larger onset potential, suggesting lower HER activity. Obviously the specific current density of the NiO/Ni/C sample is indeed higher than that of the ALD-NiO/C and Ni/C samples under a certain applied voltage, in which the current densities for the NiO/Ni/C samples of 10 mA·cm^−2^ and 100 mA·cm^−2^ are achieved at low overpotential vs. the reversible hydrogen electrodes (RHE) of 189 and 337 mV, respectively. The overpotential of NiO/Ni nanoparticles loaded at carbon fiber paper at 10 mA·cm^−2^ is smaller than most recently reported HER catalysts in alkaline solution [18,19,20,21,22,23,24,25,26], including CoNx/C (247 mV) [19], NiFe layered double hydroxide (LDH)/Ni foam (210 mV) [21], CoP/CC (209 mV) [24], and N-Co@G (337 mV) [26]. The highest HER activity of the NiO/Ni/C sample results from the NiO/Ni nanoparticles during the annealing process with reduction and mild oxidation treatment. There is little influence of carbon fiber paper for HER activity, due to its very small current density. To further investigate the HER activity, the linear portions of Tafel plots are fitted. In Figure 4b, the NiO/Ni/C shows a Tafel slope of 132 mV per decade, which outperformed the Ni/C sample of 141 mV·dec^−1^ and the ALD-NiO/C sample of 151 mV·dec^−1^. The smaller Tafel plot of NiO/Ni/C implies a more rapid HER rate for the NiO/Ni/C electrode, compared with the ALD-NiO/C and Ni/C electrode. The more active HER activity and smaller Tafel plots are attributed to the larger conductivity shown in Figure S6 and Table S1, compared with those of the ALD-NiO/C and the Ni/C samples. The electrocatalytic oxygen evolution reaction (OER) performance was also investigated in 1 M KOH (Figure 4c). Apparently the NiO/Ni/C sample affords a current density of 10 mA·cm^−2^ at an overpotential of only 257 mV, which is much lower than those of the ALD-NiO/C and Ni/C samples. It indicates that NiO/Ni nanoparticles loaded carbon fiber paper exhibit superior OER activity.

According to the OER mechanistic studies of Ni-based catalysts in alkaline electrolytes, it involves four consequent elementary steps and can be depicted as follows: [27,28]

Ni^2+^ + 3OH^−^ ↔ NiOOH + H_2_O + e^−^ (1)

NiOOH + OH^−^ ↔ NiO(OH)_2_ + e^−^ (2)

NiO(OH)_2_ + 2OH^−^ ↔ NiOO_2_ + 2H_2_O +2e^−^ (3)

NiOO_2_ + OH^−^ → NiOOH + O_2_ + e^−^ (4)

Overall OER: 4OH^−^ → O_2_ + 2H_2_O + 4e^−^ (5)

Surface Ni atoms on the catalyst surface are first partially oxidized into NiOOH, which is evidenced by the oxidation peak at around 1.51 V in Figure 4c. Then the NiOOH is further oxidized to NiOO_2_ for a NiO/Ni/C sample with higher potential, which lead to oxygen evolution. Moreover a more intense oxidation peak for NiO/Ni/C samples than those of ALD-NiO/C and Ni/C is presented in Figure 4c as well, indicating more active sites of formation on the electron conductive NiO/Ni/C sample [28]. Based on the great electrocatalytic activity of NiO/Ni/C for both HER and OER, the NiO/Ni/C can be regarded as an active electroctalyst for overall water splitting using NiO/Ni/C as cathode and anode. As shown in Figure 4d, the NiO/Ni/C||NiO/Ni/C system in a two-electrode system affords a current density of 10 mA·cm^−2^ at a cell voltage of 1.93 V, which is lower than those of Ni/C and ALD-NiO/C. Therefore both the HER and OER performances demonstrate the significant effect of the NiO/Ni nanoparticles for overall water splitting, which are loaded on carbon fiber paper using the combined treatment of ALD, reduction, and oxidation annealing processes. The stability of the NiO/Ni/C sample was measured for 10 h in 1 M KOH (Figure 4e). There was little decay during 10 h of continuous HER measurement, suggesting the greater stability and durability of the NiO/Ni sample. The HER activity of the reduced sample under the H_2_/Ar conditions at different annealing temperatures, from 300 to 600 °C, are shown in Figure S7. All of the reduced samples exhibit higher current density than the ALD-NiO/C sample, and the reduced sample annealed at 500 °C shows the lowest overpotential at a current density of 100 mA·cm^−2^ and the lowest Taple slope, compared with another reduced samples under different annealing temperatures. This is due to the lower number of nanoparticles in the 500 °C reduced sample (Figure S8), compared with the larger nanoparticles of 400 °C and 600 °C. Throughout the detailed HER activity, we summarize that the NiO/Ni nanoparticles exhibit larger surface area and conduction activity, leading to the higher current density and the lower Tafel plot.

## 4. Conclusions

In summary, we combined the ALD technique with reduction and oxidation treatments to achieve the NiO/Ni nanoparticles catalyst deposited on carbon fiber paper, which exhibits active hydrogen evolution reaction performance and high durability with no performance decay for 10 h, working in 1 M KOH. The NiO/Ni/C electrode exhibits a current density of 10 mA·cm^−2^ with an overpotential of 189 mV for the hydrogen evolution reaction. Apparently it also affords a current density of 10 mA·cm^−2^ at an overpotential of 257 mV for the oxygen evolution reaction. The significant improvement of catalytic activity is rendered by the nanoparticle-structure during the reduction and slight oxidation during the annealing treatment. Furthermore the facile synthetic procedure of the NiO/Ni/C catalyst would open up a promising way to achieve more efficient and more active water splitting for the hydrogen evolution reaction and oxygen evolution reactions.

## Supplementary Materials

The following are available online at www.mdpi.com/1996-1944/10/1/15/s1; Figure S1: The photogarph of NiO loaded on carbon fiber paper after different treatment, Figure S2: High-resolution transmission electron microscope (HRTEM) images of the ALD-NiO/C sample, Figure S3: The X-ray diffractometer (XRD) patterns of carbon paper, Figure S4: Scanning electron microscope (SEM) images and electron dispersive X-ray spectrum (EDX) spectra, Figure S5: X-ray photoelectron spectrometer (XPS) survey scan of NiO deposited on carbon fiber paper using the ALD technique, Figure S6: The electrochemical impedance spectra (EIS) of ALD-NiO/C, Ni/C and NiO/Ni/C, Figure S7: Hydrogen evolution reaction (HER) of reduced NiO/C samples under different annealing temperatures, Figure S8: SEM images of reduced NiO/C samples at different H_2_-annealing temperature, Table S1: The resistance value of equivalent circuit.
