# Supplementary material for: Enhanced Electrocatalytic Activity for Water Splitting on NiO/Ni/Carbon Fiber Paper
Source: Materials (Basel). 2016 Dec 28;10(1):15. doi: 10.3390/ma10010015 (PMC5344590; doi:10.3390/ma10010015)
Supplement: Supplementary file 1 [file materials-10-00015-s001.pdf]

Supplementary Materials: Enhanced Electrocatalytic Activity for Water Splitting on NiO/Ni/Carbon Fiber Paper

Ruoyu Zhang, Hehe Wei, Wenjie Si, Gang Ou, Chunsong Zhao, Mingjun Song, Cheng Zhang and Hui Wu

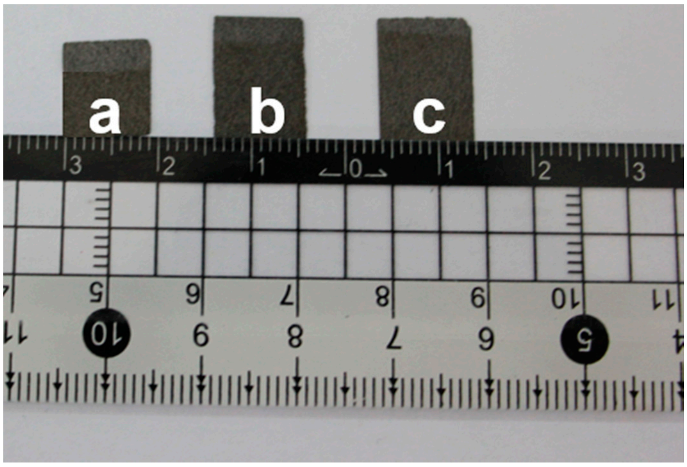

Figure S1. The photogarph of NiO loaded on carbon fiber paper after different treatment. (**a**) Atomic layer deposition (ALD)-NiO/C sample; (**b**) Ni/C sample; (**c**) NiO/Ni/C sample. The light color of practical carbon fiber paper is due to cover of clips during the ALD process. The alternate color of carbon fiber paper indicates changed samples after different treatment.

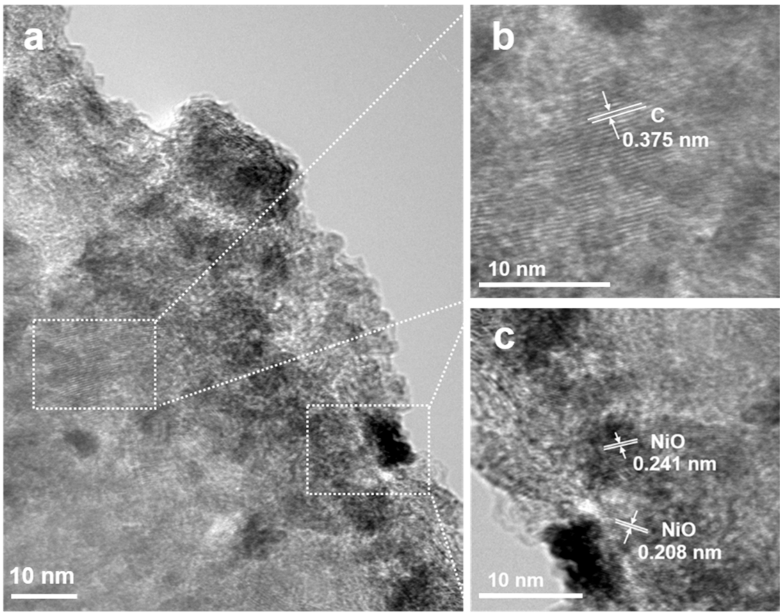

Figure S2. High-resolution transmission electron microscope (HRTEM) images of the ALD-NiO/C sample. (**b**,**c**) is a partially magnified section of (**a**).

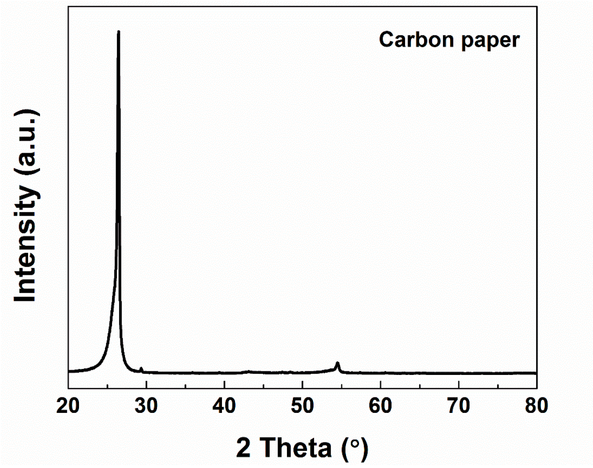

Figure S3. The X-ray diffractometer (XRD) patterns of carbon paper.

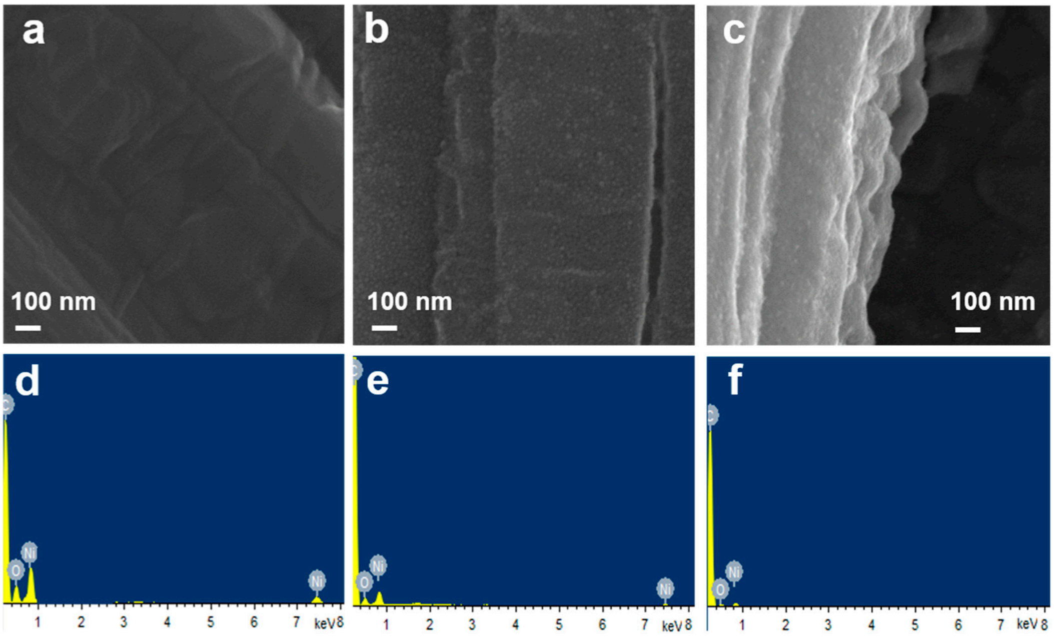

Figure S4. Scanning electron microscope (SEM) images and electron dispersive X-ray spectrum (EDX) spectra of (**a**,**d**) for the ALD-NiO/C sample; (**b**,**e**) for the Ni/C sample, as well as (**c**,**f**) for the NiO/Ni/C sample.

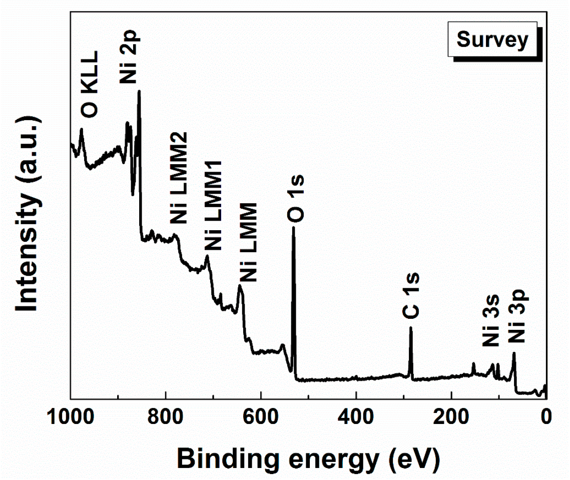

Figure S5. X-ray photoelectron spectrometer (XPS) survey scan of NiO deposited on carbon fiber paper using the ALD technique.

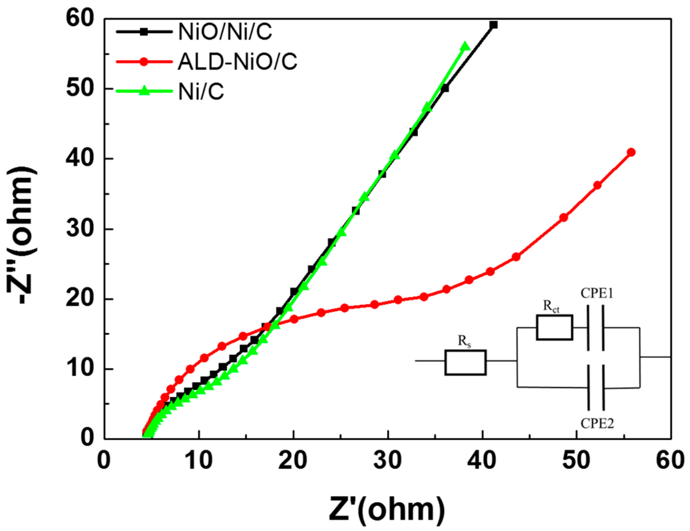

Figure S6. The electrochemical impedance spectra (EIS) of ALD-NiO/C, Ni/C and NiO/Ni/C. Z′ represents real number and Z′′ represents imaginary number.

Table S1. The resistance value of equivalent circuit. R_s represents solution resistance; R_{ct} represents reactive resistance; CPE1 and CPE2 represent capacitance.

Sample	R_s (ohm)	R_{ct} (ohm)	CPE1 (mMho)	CPE2 (μMho)
ALD-NiO/C	4.33	42.8	1.78	143
Ni/C	4.22	15.4	1.20	176
NiO/Ni/C	4.36	17.2	1.16	162

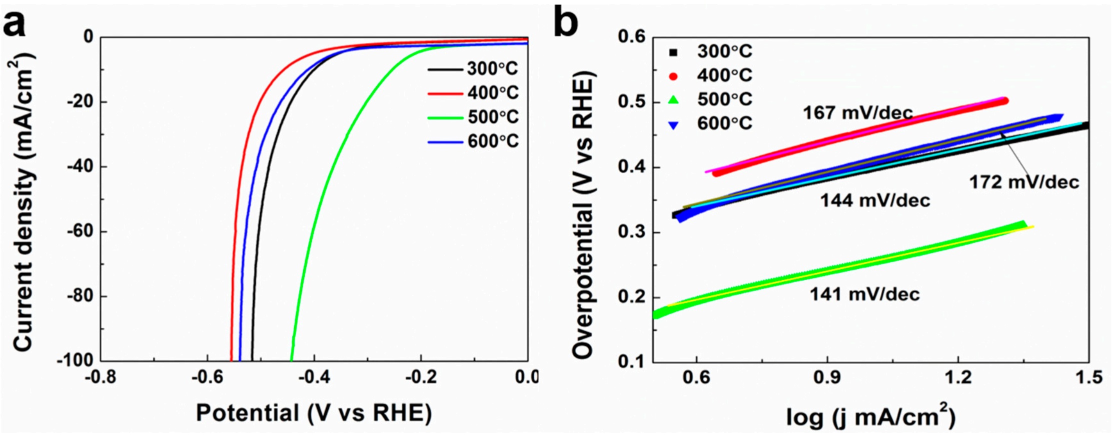

Figure S7. Hydrogen evolution reaction (HER) of reduced NiO/C samples under different annealing temperatures, from 300 to 600 °C. (**a**) Linear Sweep Voltammetry (LSV) polarization in 1 M KOH with the scan rate of 5 mV·s^{-1}; (**b**) the corresponding HER Tafel plot. All LSV curves are iR corrected. iR represents internal resistance in different solutions , it is a parameter.

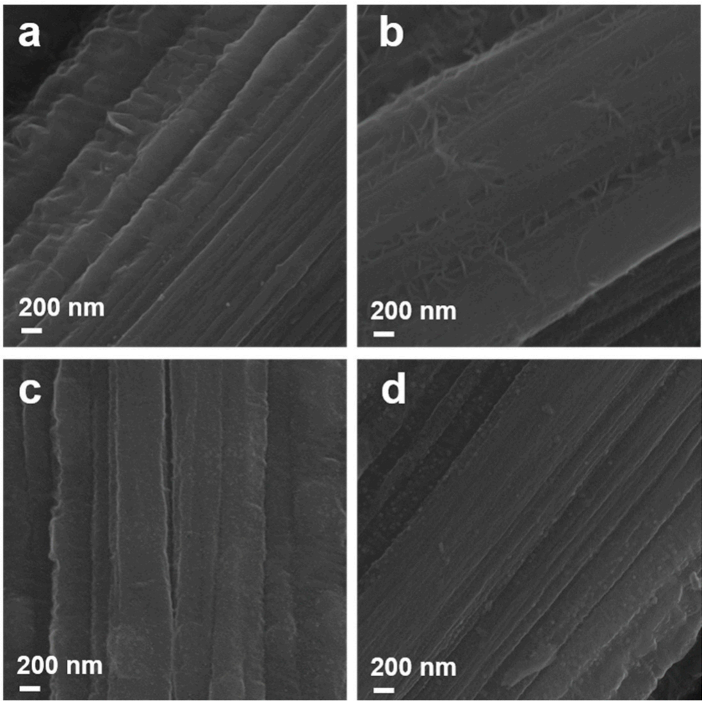

Figure S8. SEM images of reduced NiO/C samples at (**a**) 300 °C; (**b**) 400 °C; (**c**) 500 °C; and (**d**) 600 °C H_2-annealing temperature.
